# *Notes from the Field:* Administration of Expired Injectable Influenza Vaccines Reported to the Vaccine Adverse Event Reporting System — United States, July 2018–March 2019

**DOI:** 10.15585/mmwr.mm6823a3

**Published:** 2019-06-14

**Authors:** Elisabeth M. Hesse, Beth F. Hibbs, Maria V. Cano

**Affiliations:** ^1^Epidemic Intelligence Service, CDC; ^2^Division of Healthcare Quality Promotion, National Center for Emerging and Zoonotic Infectious Diseases, CDC.

Influenza vaccination is recommended annually for persons aged ≥6 months for the prevention and control of influenza (*1*). Every year, injectable inactivated influenza vaccine (IIV) has a standard expiration date of June 30 for the upcoming influenza season (i.e., July 1–June 30 of the following year). Vaccination with an expired influenza vaccine might not protect against influenza infection because different influenza virus strains can be included in the vaccine each year; in addition, protection against viruses included in the vaccine could wane if vaccine potency decreases over time. During July 11, 2018–March 29, 2019 in the United States, the Vaccine Adverse Event Reporting System (VAERS) received 125 reports of 192 patients receiving expired IIV during the 2018–19 influenza season (*2*), during which time 169.1 million doses of seasonal influenza vaccine were distributed (*3*). Dates of vaccination were documented for 102 patients and ranged from July 2, 2018, to January 16, 2019. The number of expired vaccine doses administered increased in September and decreased after October, coinciding with dates when influenza vaccine is typically given (Figure). Ages were available for 103 vaccine recipients. Seventy-three recipients (70.1%) were identified as being in high-risk age groups for influenza; eight were aged <5 years, and 65 were aged >50 years (*1*). An additional six reports specified that the patient had been pregnant at time of vaccination; pregnancy outcomes were not reported. Adverse events after the administration of an expired IIV were rarely reported (four of 125 reports; 3.2%). None were serious, and adverse events were consistent with adverse events for seasonal IIV.

**FIGURE:**
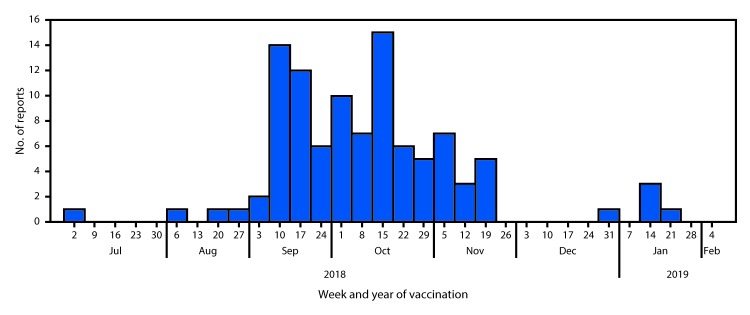
Number of reports (n = 102) with documented dates of administration of expired injectable influenza vaccine — Vaccine Adverse Event Reporting System, United States, July 2018–January 2019

The VAERS adverse event findings suggest that expired IIV does not pose additional risks for adverse events beyond those of seasonal IIV. Vaccine failure was not assessed. In most reports, factors that contributed to administration of expired vaccine were not specified; however, one cluster of reports from a pharmacy stated that four persons received expired vaccine doses that had been mistakenly shipped from another pharmacy. Seven reports detailed that patients were offered revaccination with the current season’s influenza vaccine; of these, three confirmed revaccination.

As a spontaneous reporting surveillance system, VAERS likely captures only a small fraction of expired IIV administered; therefore, this error might be more common than VAERS data indicate. CDC’s Vaccine Storage and Handling Toolkit contains guidance pertaining to prevention of and mitigation of administration of expired vaccines and is available online (https://www.cdc.gov/vaccines/hcp/admin/storage/toolkit/index.html) (*4*). Vaccine stock should be rotated and examined for expired doses regularly. Any expired vaccines and diluents should be removed immediately to avoid inadvertent administration (*4*).

Vaccines should be inspected for expiration before they are administered or transported to other facilities. Facility vaccine coordinators need to be aware of the standard expiration date of June 30 for IIV and make plans for the safe disposal or return of any remaining doses of IIV after that date. Sometimes unused vaccine may be returned for credit, even if the doses must be discarded. State immunization programs or vaccine manufacturers should be contacted to determine whether such provisions apply. Any person who receives an expired influenza vaccine should be revaccinated with the current season’s influenza vaccine.
